# Systems biology approaches to identify driver genes and drug combinations for treating COVID-19

**DOI:** 10.1038/s41598-024-52484-8

**Published:** 2024-01-26

**Authors:** Ali Ebrahimi, Farinaz Roshani

**Affiliations:** Department of Physics, Alzahra University, Tehran, Iran

**Keywords:** Computational biology and bioinformatics, Systems biology, Physics

## Abstract

Corona virus 19 (Covid-19) has caused many problems in public health, economic, and even cultural and social fields since the beginning of the epidemic. However, in order to provide therapeutic solutions, many researches have been conducted and various omics data have been published. But there is still no early diagnosis method and comprehensive treatment solution. In this manuscript, by collecting important genes related to COVID-19 and using centrality and controllability analysis in PPI networks and signaling pathways related to the disease; hub and driver genes have been identified in the formation and progression of the disease. Next, by analyzing the expression data, the obtained genes have been evaluated. The results show that in addition to the significant difference in the expression of most of these genes, their expression correlation pattern is also different in the two groups of COVID-19 and control. Finally, based on the drug-gene interaction, drugs affecting the identified genes are presented in the form of a bipartite graph, which can be used as the potential drug combinations.

## Introduction

After more than three years since the beginning of the COVID-19 epidemic, we still do not have a complete understanding of the disease process, early diagnosis, providing appropriate treatment and dealing with new strains of the disease. In addition to the many deaths, it has had during this period, on the one hand, due to its effect on other diseases, COVID-19 is considered a serious risk to public health^1–4^. And on the other hand, it has also challenged the social, cultural, and economic balances of societies^5–9^. Therefore, in order to provide treatment solutions, many databases have been formed and various bioinformatics researches have been conducted and various results have been provided^10–15^. But due to the unknown nature of the disease and the different goals and tools of the researches, the presented results of the processes and genes involved in the disease cover a wide range. Therefore, it is necessary to carry out research that is based on comprehensive analysis and integration of the results, by using the systems biology approaches based on the types of their effects on each other, to find a useful and comprehensive treatment method. Mostly, the research that considers the role of each gene in the progression of the disease separately and without its interactions with genes and other biomolecules, compared to the methods that consider the set of factors and their effects on each other in the form of complex networks and by using different network analysis tools, they seek to determine the role of each factor in the formation and progression of the disease, are less efficiency and have more side effects^16–23^.

Network science is an emerging field that represents existing complex systems in the form of networks, where system components are considered as vertices and connections between components as network edges. And it has become a powerful conceptual model in the field of computational biology for understanding biological relationships at the system level^24–29^. In the analysis of complex networks depending on the type and size of the network as well as the purpose of the research, various analytical tools from graph theory are available to determine important vertices and edges, identify motifs and modules, and finally infer general parameters of the network and compare it in different modes. One of the widely used tools in network analysis is checking controllability and identifying driver vertices^30–33^.

In order to determine the drug targets, one of the most practical methods is the use of controllability algorithms in the network with the aim of finding vertices with the least number in the network and strategies to change from an unfavorable state (disease) to a favorable state (Health), by using the intervention like medicine and other therapeutic agents. Several researches have been conducted with the perspective of system biology and various analyzes of the obtained bioinformatic networks, including checking the controllability and identifying driver vertices in order to obtain a collection of important and effective genes in COVID-19^34–37^.

In addition to network analysis of the omics data and identification of the effective and driver vertices to provide therapeutic solutions related to COVID-19, several studies have been conducted based on machine learning methods to identify signatures and the rules between them to diagnose COVID-19. For example, in an article, multiple machine learning methods on transcriptomics data of upper airway tissue with acute respiratory illnesses led to the identification of effective qualitative biomarkers and quantitative rules for distinguishing COVID-19 patients from other infectious^38^. In another work, the methylation data is first analyzed using the Monte Carlo feature selection method to obtain a feature list, and a decision tree is used to identify methylation features and decision rules that clearly distinguish different cases^39^. In an article, key methylation sites that have distinct patterns among patients with COVID-19 at different ages, have been identified^40^. Also, in another work, the biomarkers related to COVID-19 have been classified according to the severity of the disease^41^, microRNA, methylation signatures, and the rules between them that determine the severity of COVID-19 in different patients have been identified based on machine learning methods^42,43^.

In this manuscript; first, with the aim of considering the results of various researches conducted in order to obtain genes related to COVID-19, genes that have been reported with a valid *p* Value or with a large number of references in different articles, Collected. Then, by forming a PPI network between related proteins, centrality analysis has been performed and proteins that have many connections have been identified. Next, by targeting these proteins, in the directed network of signaling pathways related to COVID-19, the vertices that have the highest control power have been determined. Then, with correlation and DEG analyzes in the expression data, it has been shown that the identified hub and driver genes mainly in the two groups of COVID-19 and control have significant expression differences and secondly, their expression correlation patterns change. In the end, by using the connections between the existing drugs and the set of genes obtained, the bipartite drug-gene network, the drugs affecting each of the obtained genes have been determined, which could be potential combinations of existing drugs in order to repurpose drugs to treat COVID-19.

## Results

### Collection of genes related to COVID-19 based on literature

In the first stage, the set of genes related to corona disease have been collected based on the literature. For this purpose, two databases; the CORMINE Medical online and DisGeNET have been used. In the CORMINE database, the set of related genes is ranked based on the *p* Value criteria. *564* genes with *p* Value less than *0.05* were selected (Table S1). The DisGeNET database has ranked the set of related genes based on the number of references. Genes with more than *5* references have been selected, which is equal to *296* genes (Table S2). Due to the commonality of some genes, *757* genes related to COVID-19 that have many references or small *p* Value were collected from the above two sets, which were used for further analysis.

### PPI network construction and analysis

**Figure 1:**
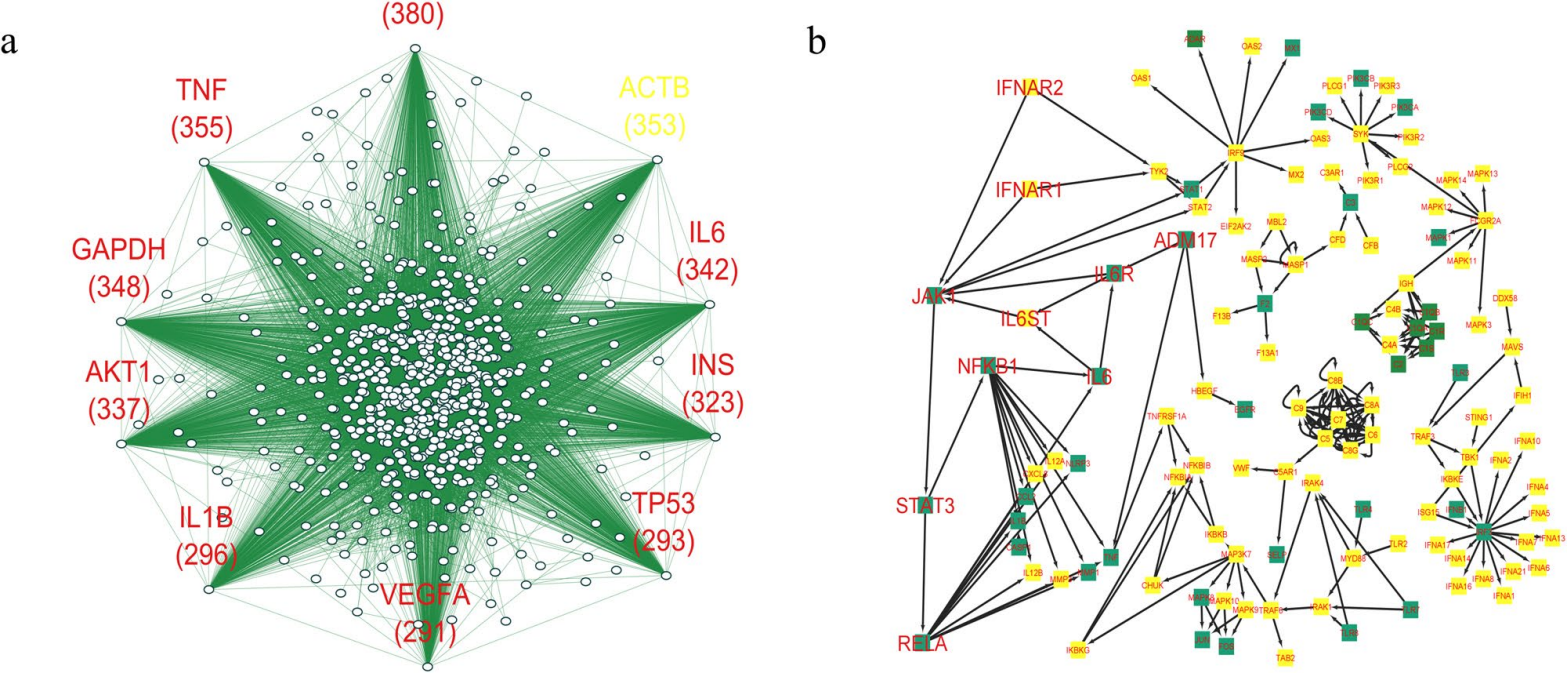
Bioinformatic networks related to COVID-19. (**a**) Undirected PPI network obtained from the STRING database between the identified proteins related to COVID-19 based on the literature. The *10* proteins with the highest degree are identified. ACTB protein has no interaction with COVID-19 proteins. (**b**) Directed network related to the signaling pathways of COVID-19 obtained from the KEGG database. Common vertices with the set of vertices with a degree greater than 50 in the PPI network and interacting with COVID-19 proteins are considered as control targets. (Green vertices). The *10* driver vertices with the highest control power for the considered target are specified in the network (written in a larger format).

In the following, among the collected genes related to COVID-19, genes that have many connections and effects on other genes have been identified. For this purpose, the PPI network between the corresponding proteins is formed based on the STRING database in which, functional and structural relationships is considered (Fig. 1a). The *10* proteins with the highest degree in the constructed network are indicated in the figure along with the number of connections. Also, the set of all proteins with a degree greater than *50* have been identified.

Next, it has been investigated that the identified hub proteins necessarily interact with the COVID-19 proteins. For this purpose, the human genes associated with COVID-19 which were obtained based on the virus-human protein interaction network and using the gene ontology, have been used^44^. Out of 10 identified hub proteins, 9 proteins have interactions which are considered for further analysis. (ACTB protein has no interaction) Also, out of 310 proteins with a degree higher than 50, 239 proteins have interaction and 71 proteins have no interaction, which are categorized into two groups in Table S3. A set of 239 proteins with a degree of more than 50 and having interaction, are considered control targets in signaling pathways related to COVID-19.

### Controllability of signaling pathways related to COVID-19

Because the directional network related to the signaling pathways of COVID-19 includes the effect and effectiveness relationships between the genes in the network and specifies functional relationships that different genes have on each other during the progression of the disease. To analyze the controllability, the signaling pathways in the KEGG database^45^ have been used. At this stage, the directed network related to the signaling pathways of COVID-19 has been extracted. (Coronavirus disease—COVID-19—Homo sapiens) (Fig. 1b) Next, common vertices are obtained in the directed network, and the set of proteins with a degree higher than 50 and interacting with COVID-19 proteins of the previous step is considered as the control target. (Vertices that marked with green color) and then based on the target controllability algorithm with the least mediator vertices^33^, *10* vertices that have the most power to control the target set have been determined (the set of driver vertices is marked in the figure).

Because each of the obtained vertices, according to the definition of the vertices with the highest control power, must control a large number of the set of target vertices, and on the other hand, each of the vertices of the target set has many connections in the PPI network, so the obtained driver vertices should affect the set of many proteins in the PPI network between the proteins related to COVID-19.

*IL6* protein is among the top *9* proteins in the PPI network and is also among the top *10* drivers in signaling pathways. Therefore, a total of *18* proteins have been considered as a set of hub and driver proteins for further analysis. Driver and hub genes identified, have subscription with the results of machine learning methods to identify signatures associated with COVID-19. For example, the TNF gene is one of the essential biomarkers of COVID-19^38^, or TNF and INS genes have been obtained as transcriptional biomarkers for severe COVID-19 by machine learning methods^41^.

### Analysis of expression data

**Figure 2:**
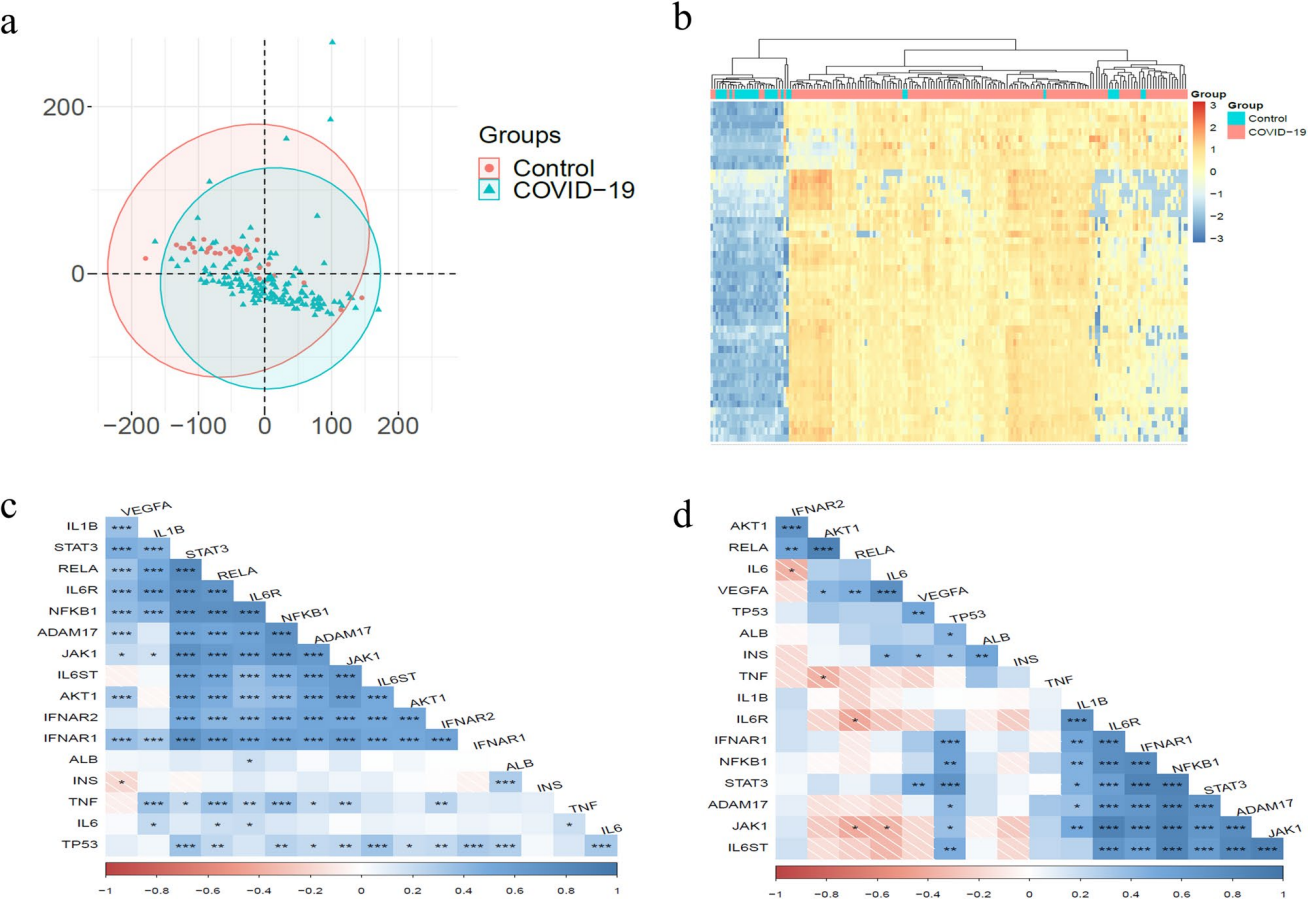
Expression data analysis results (**a**) Principal component analysis in two groups of COVID-19 and control. (**b**) Heatmap of 50 genes with the most expression changes. (**c**) Expression correlation of hub and driver genes in the group of COVID-19. (**d**) Expression correlation of hub and driver genes in control group.

In the following, to evaluate the obtained results, analysis of expressive data has been done. For this purpose, expression data set *GSE163151* was used, in which *404* expression profiles were collected from nasal swabs and blood of healthy people and people suffering from COVID-19 and other viral and bacterial infections^46^. Of the total data, *31* data profiles are from healthy people (control group) and *145* data profiles are from people with COVID-19. First, DEG analysis has been performed between two control and COVID-19 groups. Figure 2a,b show the Principal component analysis in two groups and the heatmap of *50* genes with the highest expression changes^15^. Then the co-expression analysis between the hub and driver genes obtained in the previous steps has been performed. Figures 2c,d show the level of expression correlation in the COVID-19 group and the control group, respectively. The results show the correlation changes in gene expression in the two groups. In general, the correlation is higher in the COVID-19 group.

Of the *18* selected hub and driver genes, *13* genes with a *p* Value less than *0.05* have expression differences in the two groups of COVID-19 and control, as shown in Table 1. Also, AveExpr and Log2FC of each gene are expressed in the second and third columns respectively.

**Table 1:** The set of hub and driver genes with *P* Value less than 0.05 in the two groups of COVID-19 and control have expression differences.

Gene symbol	AveExpr	Log2FC	*p* value
IFNAR1	3.3578	0.4686	0.0000
VEGFA	3.5261	− 2.1858	0.0000
STAT3	4.4286	− 0.728	0.0002
IL6ST	3.1431	0.5109	0.0007
IL6R	2.7561	0.7218	0.0016
RELA	3.251	− 0.6137	0.0037
IL6	0.8597	− 0.4065	0.0051
JAK1	4.0955	0.3492	0.0058
IFNAR2	2.6462	0.3756	0.0075
TNF	1.5162	− 0.5443	0.0091
AKT1	2.7849	0.2576	0.0217
IL1B	2.336	− 0.6236	0.0286
TP53	2.1182	0.2761	0.0361

### Identification of drugs related to the obtained gene set

**Figure 3:**
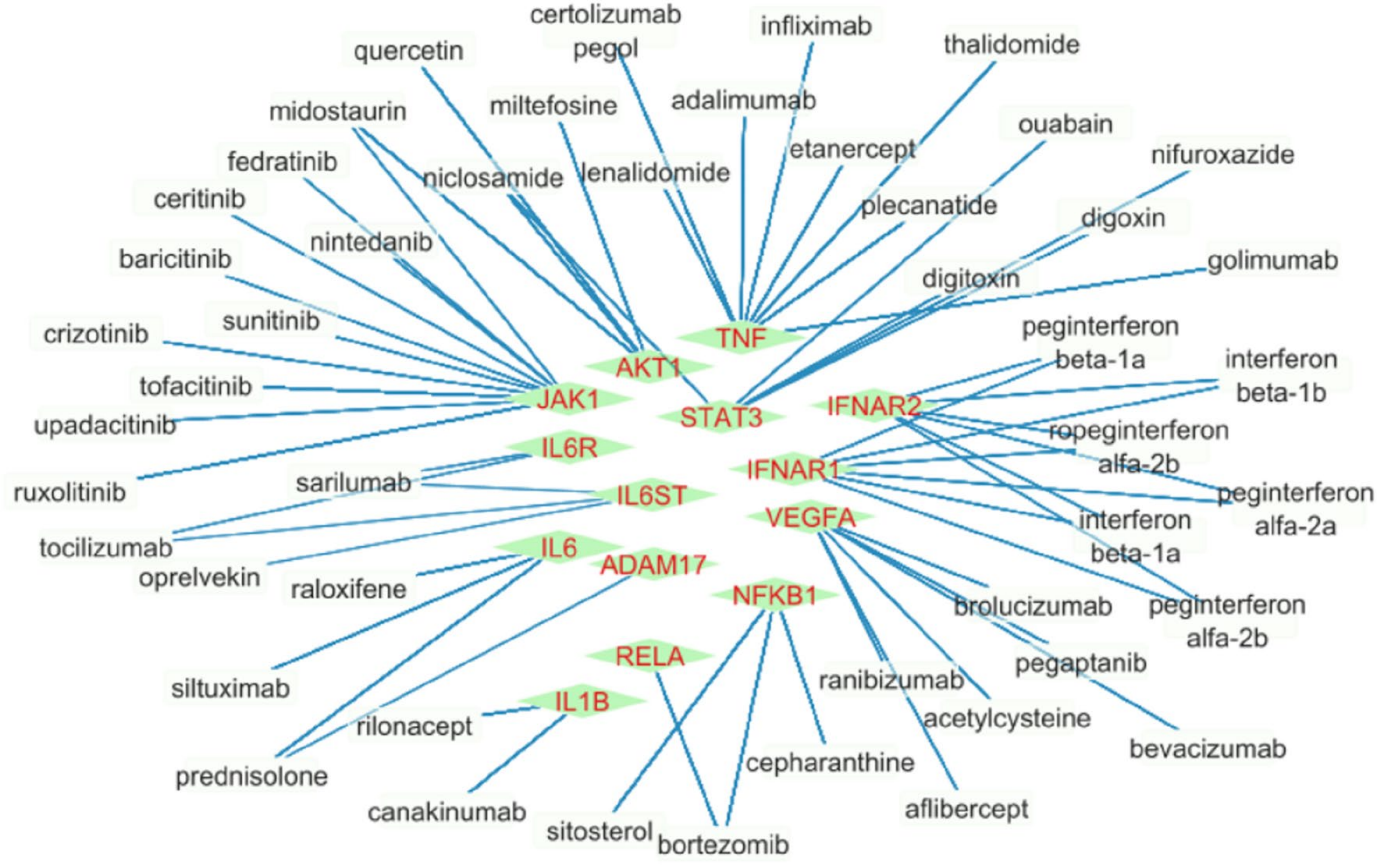
Bipartite network of connections between genes and drugs.

In order to identify potential drug combinations against COVID-19, existing drugs that affect each of the identified hub and driver genes based on DrugCentral^47^ and KEGG pathways were presented as potential drug targets. The related genes and drugs are indicated in the bipartite graph format shown in Fig. 3. In the following, Similar to the process used to find candidate drugs for lung cancer^48^, based on the STITCH database, which identifies chemical-chemical and chemical-proteins interaction, five chemical compounds corresponding to each of the 18 driver and hub genes have been determined, which are presented in figures S1 and S2.

There is considerable evidence of relationships between the identified drugs and Remdesivir, which is a well-known and widely used antiviral medicine that works by stopping the virus that causes COVID-19. including: The metabolism of Remdesivir can be decreased when combined with Midostaurin^49^. The metabolism of Bortezomib can be decreased when combined with Remdesivir^49^. The metabolism of Digitoxin can be decreased when combined with Remdesivir^49^. The excretion of Raloxifene can be decreased when combined with Remdesivir^50^. The metabolism of Sunitinib can be decreased when combined with Remdesivir^49^. The metabolism of Ceritinib can be decreased when combined with Remdesivir^49^. The metabolism of Crizotinib can be decreased when combined with Remdesivir^49^. The metabolism of Ruxolitinib can be decreased when combined with Remdesivir^49^. The metabolism of Tofacitinib can be decreased when combined with Remdesivir^51^. The serum concentration of Remdesivir can be increased when it is combined with Fedratinib^52^. The metabolism of Nintedanib can be decreased when combined with Remdesivir^49^. Details of each of the 61 drug-gene interactions identified in Fig. 3 between 14 genes and 48 drugs are specified in Table S4. Where the signaling pathways and mechanism of drug action on the relevant gene, structure id, target class, accession, action value, and action type, are specified.

## Conclusion

Due to the importance of finding a treatment solution for COVID-19, various omics data related to it have been created. Also, many bioinformatics researches have been conducted, which depending on the data selection and analysis method, have produced sometimes different results. Therefore, in the direction of a comprehensive analysis, it is necessary to consider all the presented results.

In this paper, to create a PPI network related to COVID-19, using the results presented in different articles, a set of genes has been selected that have either a large number of references confirming its importance with COVID-19 or based on the criterion *p* Value is very important in the development of COVID-19. In the PPI network, which includes the functional and physical connections of the selected proteins, the vertices with the highest degree are selected. Therefore, the change in the concentration of obtained proteins will affect a large number of proteins related to COVID-19. Also, the controllability analysis of the directed network related to the signaling pathways of COVID-19 has led to the identification of driver vertices. Network control methods look for vertices in the network that have a stimulating role and when they are affected by external signals such as drugs or any other treatment method, they can hierarchically affect all network vertices and create the desired change. The above analyzes were not performed independently. Rather, the control target vertices are proteins that have many connections in the PPI network. Since the high control power is due to the presence of long paths and the high degree is due to many connections, the high control power of the vertex and the high degree of the vertex in the network are complementary to each other. Therefore, when the controlled vertices have a high degree, the influence of the stimulating vertices in the network will be doubled.

In order to evaluate and check the significance of the obtained results, expression data analysis has been done. The results confirm that the expression of the set of genes obtained is mainly different in the two groups of COVID-19 and control, and on the other hand, the correlation of the expression of pairs of genes is different in the two groups. Finally, the most important drugs that affect each of the obtained genes are shown in the form of a bipartite network, which can potentially provide new drug suggestions for the treatment of COVID-19.

The process carried out in this paper to provide drug combinations for COVID-19, Collects and filters disease-related genes based on literature. Then it performs centrality and controllability analysis on different PPI networks and the related signaling pathways to identify hub and driver genes. In the following based on the analysis of expression data, the obtained results are evaluated. Finally, based on various drug-gene relationships, appropriate drug combinations are suggested, which can be a general pipeline that can be considered for any other disease.

## Material and methods

### Hub vertices selection

Determination of hub vertices in the PPI network is based on the degree centrality criterion. The 10 proteins with the highest degree have been selected as the 10 hub proteins.

### Controllability of complex networks

A dynamical system is controllable if, by entering appropriate input signals, the state of vertices can be transferred from any position to any desired position with a finite number of steps. Checking the exact controllability is based on the Kalman's controllability rank condition. On the other hand, checking the structural controllability is based on the minimum input theorem, which determines the relationship between maximum matching and driver vertices based on the Lin's structural controllability theorem.

### Controllability with minimum mediator

The target controllability algorithm with minimum mediator, which is based on the length of the paths between vertices in the network, identifies driver vertices that can control the desired target with the least number of intermediary vertices.

### Control centrality

As a centrality measure, it seeks to identify the vertices that control the largest number of vertices in the whole network or desired target. 10 vertices with the highest control power in the directed network related to the KEGG pathway signaling have been identified.

### Supplementary Information

Supplementary Information 1.

Supplementary Information 2.

## Data Availability

All data generated or analysed during this study are included in this published article [and its supplementary information files].

## References

[1] Baral S (2022). Competing health risks associated with the COVID-19 pandemic and early response: A scoping review. PLoS One.

[2] Connor J (2020). Health risks and outcomes that disproportionately affect women during the Covid-19 pandemic: A review. Soc. Sci. Med..

[3] Moreno C (2020). How mental health care should change as a consequence of the COVID-19 pandemic. Lancet Psychiatry.

[4] Gavin B, Lyne J, McNicholas F (2020). Mental health and the COVID-19 pandemic. Ir. J. Psychol. Med..

[5] Ratten V (2020). Coronavirus (covid-19) and entrepreneurship: Changing life and work landscape. J. Small Bus. Entrep..

[6] Cheer JM (2020). Human flourishing, tourism transformation and COVID-19: A conceptual touchstone. Tour. Geogr..

[7] Ratten V (2021). Coronavirus (Covid-19) and entrepreneurship: Cultural, lifestyle and societal changes. J. Entrepreneurship Emerg. Econ..

[8] He H, Harris L (2020). The impact of Covid-19 pandemic on corporate social responsibility and marketing philosophy. J. Bus. Res..

[9] Mofijur M (2021). Impact of COVID-19 on the social, economic, environmental and energy domains: Lessons learnt from a global pandemic. Sustain. Product. Consum..

[10] Stasi C, Fallani S, Voller F, Silvestri C (2020). Treatment for COVID-19: An overview. Eur. J. Pharmacol..

[11] Li X (2021). Network bioinformatics analysis provides insight into drug repurposing for COVID-19. Med. Drug Discov..

[12] Li R, Li Y, Liang X, Yang L, Su M, Lai KP (2021). Network Pharmacology and bioinformatics analyses identify intersection genes of niacin and COVID-19 as potential therapeutic targets. Brief. Bioinform..

[13] Aghdam R, Habibi M, Taheri G (2021). Using informative features in machine learning based method for COVID-19 drug repurposing. J. Cheminform..

[14] Masoudi-Sobhanzadeh Y, Esmaeili H, Masoudi-Nejad A (2022). A fuzzy logic-based computational method for the repurposing of drugs against COVID-19. BioImpacts BI.

[15] Zhang W (2022). COVID19db: A comprehensive database platform to discover potential drugs and targets of COVID-19 at whole transcriptomic scale. Nucleic Acids Res..

[16] Kitano H (2002). Computational systems biology. Nature.

[17] Vandamme D, Minke BA, Fitzmaurice W, Kholodenko BN, Kolch W (2014). Systems biology-embedded target validation: Improving efficacy in drug discovery. Wiley Interdiscipl. Rev.: Syst. Biol. Med..

[18] Kinnings SL, Liu N, Buchmeier N, Tonge PJ, Xie L, Bourne PE (2009). Drug discovery using chemical systems biology: Repositioning the safe medicine Comtan to treat multi-drug and extensively drug resistant tuberculosis. PLoS Comput. Biol..

[19] Prathipati P, Mizuguchi K (2016). Systems biology approaches to a rational drug discovery paradigm. Curr. Top. Med. Chem..

[20] Bugrim A, Nikolskaya T, Nikolsky Y (2004). Early prediction of drug metabolism and toxicity: Systems biology approach and modeling. Drug Discov. Today.

[21] Davidov E, Holland J, Marple E, Naylor S (2003). Advancing drug discovery through systems biology. Drug Discov. Today.

[22] Arrell D, Terzic A (2010). Network systems biology for drug discovery. Clin. Pharmacol. Ther..

[23] Wagner HJ, Weber W, Fussenegger M (2021). Synthetic biology: Emerging concepts to design and advance adeno-associated viral vectors for gene therapy. Adv. Sci..

[24] Pavlopoulos GA (2011). Using graph theory to analyze biological networks. BioData Min..

[25] Aghdam R, Alijanpour M, Azadi M, Ebrahimi A, Eslahchi C, Rezvan A (2016). Inferring gene regulatory networks by PCA-CMI using Hill climbing algorithm based on MIT score and SORDER method. Int. J. Biomath..

[26] Ma'ayan A (2009). Insights into the organization of biochemical regulatory networks using graph theory analyses. J. Biol. Chem..

[27] Giuliani A, Krishnan A, Zbilut JP, Tomita M (2008). Proteins as networks: Usefulness of graph theory in protein science. Curr. Protein Pept. Sci..

[28] Kantelis KF (2022). Graph theory-based simulation tools for protein structure networks. Simulat. Modell. Pract. Theory.

[29] Zhou Z, Guang H (2023). Applications of graph theory in studying protein structure, dynamics, and interactions. J. Math. Chem..

[30] Liu Y-Y, Slotine J-J, Barabási A-L (2011). Controllability of complex networks. Nature.

[31] Yuan Z, Zhao C, Di Z, Wang W-X, Lai Y-C (2013). Exact controllability of complex networks. Nat. Commun..

[32] Gao J, Liu Y-Y, D'souza RM, Barabási A-L (2014). Target control of complex networks. Nat. Commun..

[33] Ebrahimi A, Nowzari-Dalini A, Jalili M, Masoudi-Nejad A (2020). Target controllability with minimal mediators in complex biological networks. Genomics.

[34] Popescu V-B, Kanhaiya K, Năstac DI, Czeizler E, Petre I (2022). Network controllability solutions for computational drug repurposing using genetic algorithms. Sci. Rep..

[35] Guo W-F, Zhang S-W, Feng Y-H, Liang J, Zeng T, Chen L (2021). Network controllability-based algorithm to target personalized driver genes for discovering combinatorial drugs of individual patients. Nucleic Acids Res..

[36] Habibi M, Taheri G, Aghdam R (2021). A SARS-CoV-2 (COVID-19) biological network to find targets for drug repurposing. Sci. Rep..

[37] Sharma A, Cinti C, Capobianco E (2017). Multitype network-guided target controllability in phenotypically characterized osteosarcoma: role of tumor microenvironment. Front. Immunol..

[38] Zhang Y-H (2021). Identifying transcriptomic signatures and rules for SARS-CoV-2 infection. Front. Cell Dev. Biol..

[39] Li Z (2022). Identifying methylation signatures and rules for COVID-19 with machine learning methods. Front. Mole. Biosci..

[40] Chen L (2022). Identification of DNA methylation signature and rules for SARS-CoV-2 associated with age. Front. Biosci. -Landmark.

[41] Li X (2022). Identification of transcriptome biomarkers for severe COVID-19 with machine learning methods. Biomolecules.

[42] Ren J, Guo W, Feng K, Huang T, Cai Y (2022). Identifying MicroRNA markers that predict COVID-19 severity using machine learning methods. Life.

[43] Liu Z (2022). Identification of methylation signatures and rules for predicting the severity of SARS-CoV-2 infection with machine learning methods. Front. Microbiol..

[44] Zhang Y, Zeng T, Chen L, Ding S, Huang T, Cai Y-D (2020). Identification of COVID-19 infection-related human genes based on a random walk model in a virus–human protein interaction network. BioMed Res. Int..

[45] Kanehisa M, Goto S (2000). KEGG: Kyoto encyclopedia of genes and genomes. Nucleic Acids Res..

[46] Ng DL (2021). A diagnostic host response biosignature for COVID-19 from RNA profiling of nasal swabs and blood. Sci. Adv..

[47] Ursu O (2016). DrugCentral: Online drug compendium. Nucleic Acids Res..

[48] Lu J (2016). Identification of new candidate drugs for lung cancer using chemical–chemical interactions, chemical–protein interactions and a K-means clustering algorithm. J. Biomol. Struct. Dyn..

[49] Zhou S-F (2008). Drugs behave as substrates, inhibitors and inducers of human cytochrome P450 3A4. Curr. Drug Metab..

[50] Karlgren M, Ahlin G, Bergström CA, Svensson R, Palm J, Artursson P (2012). In vitro and in silico strategies to identify OATP1B1 inhibitors and predict clinical drug–drug interactions. Pharm. Res..

[51] Dowty ME (2014). The pharmacokinetics, metabolism, and clearance mechanisms of tofacitinib, a janus kinase inhibitor, in humans. Drug Metab. Dispos..

[52] Ogasawara K (2020). Assessment of effects of repeated oral doses of fedratinib on inhibition of cytochrome P450 activities in patients with solid tumors using a cocktail approach. Cancer Chemother. Pharmacol..

