# Supplementary material for: Systems biology approaches to identify driver genes and drug combinations for treating COVID-19
Source: Sci Rep. 2024 Jan 26;14:2257. doi: 10.1038/s41598-024-52484-8 (PMC10817985; doi:10.1038/s41598-024-52484-8)
Supplement: Supplementary file 2 — Supplementary Information 2. [file 41598_2024_52484_MOESM2_ESM.docx]

Supplementary

Systems Biology Approaches to Identify Driver Genes and Drug Combinations for Treating COVID-19

**Ali Ebrahimi^1^, Farinaz Roshani^1*^**

**1.** Department of Physics, Alzahra University, Tehran, Iran

***Corresponding Author:**

**Farinaz Roshani, PhD.,**

Department of Physics, Alzahra University, Tehran, Iran.

**Email:** [farinaz@alzahra.ac.ir](mailto:farinaz@alzahra.ac.ir)

Tel: +98-912-435-7324

Table S.1: COVID-19 related genes with P-Value less than 0.05 (based on CORMINE database)

| P-Value | **Genes** |
| --- | --- |
| < 0.01 | ACE2, TMPRSS2, CDSN, CRP, FURIN, IL6, SH2D3C, PSMD1, IGHV3-53, SH2D3A, IL1RN, BSG, VTN, SPECC1, F2, TP53, LZTFL1, IL6R, PORCN, ACE, DPP4, BCL2, BMND7, BMND8, CLEC4M, CENPJ, CAT, TMPRSS4, CLEC4G, CTSL, CASP3, CXCL10, FUT3, TRBV11-2, TNF, IFITM3, SLC6A19, ARCN1, COPD, DNAH8, SLC6A20, RTN2, TMEM41B, NAA50, APP, INS, BCAR3, IFNAR2, ADAMTS13, IFNG, NRP1, PRL, FN1, VEGFA, ENDOU, PRAME, TSC1, PPP1CA, NELL1, C11orf71, FAM214A, FGF14-IT1, CYCSP8, F8A2, RPL6P27, SNORA81, MIR3142, LOC100506985, ARHGAP26-AS1, BSN-AS1, CDKN1A, DNAH7, TXK, CYCS, TMPRSS11D, NFKB1, ERBB2, DECR1, AAK1, CTSB, ADAM17, PTH, NOS2, CA1, SST, LEP, JUN, EGFR, CDH1, VIM, OAS2, GCG, APOL1, ACHE, BDNF, KLK3, PARP12, POMC, CTPP, MMP9, PRNT, H3F3AP6, MMP2, RGAG4, TCEAL1, TF, PPP6R3, IGF1, IGHV3-15, CCND1, OAS1, EPO, MPP5, CDKN2A, ESR1, WNK2, IFITM2, CD34, PF4, GLB1, BGLAP |
| 0.01 $\ll$ …  < 0.02 | CALM2, CALM3, TH, CALM1, ADIPOQ, NOS3, TXNRD1, SLC3A2, CD44, GFAP, PTEN, FOS, CYP19A1, BCHE, BRAF, FGF2, NLRP3, HSD17B6, DNAH7, TLR7, AFP, CTNNB1, IGKV1-9, FBXO34, SNORD35B, SNORA40, SNORA81, MIR1202, KRBOX1-AS1, GS1-24F4.2, ARHGAP26-AS1, CACNA1C-IT3, GRM7-AS3, PANO1, DPP9, TMEM41B, IFITM1, EDN1, IGHV3-66, ZDHHC20, INSR, BRCA1, SKAP2, CYP4F3, DDX58, GAST, DIANPH, NPY, TMEM27, ELN, CASP9, MTOR, CA3, KIT, APOE, CD40, ZYG11B, BCL2L1, PRNP, OAS3, YWHAZ, RET, BDNF-AS, PLB1, PLA2G1B, GSR, PARP1, CD4, TAC1, ABCB1, MPO, LOC100508689, MPD3, TMPRSS11A, NR4A1, COL11A2, PROC, ERN2, RAD51, AVP, NTF3, PCNA, CXCR4, DES, AKR1A1, SYT1, TXN, LPL, APOB, TYR, IL2, POU5F1, KDR, WNK1, FOXP3, COX18, SP1, IGHV1-24, FAM214A, ATHL1, TMPRSS11B, R52N2, SNORA18, BRCA2, TG, DAND5, PPARG, EGF, PSG1, ABCG2, IL4, RNASE1, TNFSF11, CREB1, NOS1, IFIH1, FYCO1, XCR1 |
| 0.02 $\ll$  ...  < 0.05 | TNNI3, HTR1A, SLC9A6, PARP14, TIMP1, PTK2B, SOX2, MBP, SYP, IGF2, TERT, TMPRSS11D, CASP8, PNMA2, LDOC1L, NBL1, RAC1, MDM2, PLG, CXCR6, MMP1, CXCR1, MGAM, GAPDH, APOBEC4, WASH1, STAT3, PRKAA1, GJA1, CDK2, PRKAA2, FASLG, TGFB1, SQSTM1, RB1, DMD, PRKAB1, IFI27, ARTN, IFIT1, DNASE1, CP, CXCL8, RELA, ALB, CHAT, RSAD2, PARP12, CNR1, PECAM1, DEAR, MUC16, CDK1, LDLR, SPP1, ITGAM, CPQ, NOTCH1, ARL17A, DCAKD, ELMSAN1, TTTY10, GPR141, TMEM189-UBE2V1, EGOT, CASC18, CD209, MPP5, PARK2, HSP90AA1, MLH1, ACPP, MET, TPCN2, GH1, BTK, SLC6A4, HIF1A, NES, HMGCR, SIRT1, TWIST1, ITGB2, PIKFYVE, CD86, SOD1, PIK3CA, AGTR2, SMAD3, PPARA, CYP1A1, PROS1, RHOA, HTR2A, CCL7, ITIH4, KHDRBS1, PTGS2, HP, AR, CD177, CYBB, HLA-DRB1, PLAU, VIP, NF1, HRAS, ADA, DHFR, TBC1D9, CD2, RPP30, NUP62, MMP3, TFRC, CFTR, FGFR2, SOD2, SMAD2, MAP1LC3A, ENO2, CDH17, PTGS1, CXCL12, CAV1, IL10RB, INHBE, PSEN1, CDK4, IL3, PROM1, CDC42, PPP6R2, CCR9, HSPD1, KITLG, NBR1, GDNF, SLC2A1, IGHV3-15, TRBV27, MS4A14, CCDC58, TMPRSS12, MMP13, CHGA, NQO1, IGFBP3, ISG20, IRS1, CTGF, MME, MTHFR, MYCN, COMT, SLC6A3, THM, FASN, FHIT, CYP2E1, OCLN, CYP2C19, IL1F10, NRAS, PDYN, IFNA1, MAS1, MSH2, CTLA4, IGHV1-2, ENG, SOX9, ERICD, NTS, EZH2, APOA1, MAPT, RIMS2, IFNL1, TLR8, ALOX5, NELFE, NT5E, CCNA2, CTSD, HTT, SHBG, CD80, HRASLS, MBD2, MUL1, PTPRC, MT-CO1, TNS1, HCRT, NR3C1, ENAH, G6PC, ZNF645, CHTOP, KLRC1, FLT3, PTPN11, MARK2, AKT1, HSPB1, HBB, TRIM4, ZCCHC16, LTBR, KLK4, TRPV1, TIMP2, NDUFA2, STAT5B, ZER1, ZDHHC4, MRPL24, CFAP57, TMEM64, SAPCD1, MIR510, HRCT1, ARL17B, GRIN2B, CD14, IRF3, PTBP1, CHDM, ASPG, CYP1A2, PRSS57, CYP2C9, SETMAR, CLUAP1, IGHV3-30, C15orf48, FCRL5, PTHLH, ACTB, SUMF2, GHRH, MT-CYB, HDAC9, SNCA, ITGAL, HMOX1, HLA-B, IL2RG, SMAD4, ANXA5, OR7E66P, FAS, IGF1R, MCL1, KIAA0319, AZON, FOXP4-AS1, NCAM1, FPR1, KRT19, STAT5A, GCK, HPRT1, MAP2, CD36, MECP2, EGR1, HGF, ZGLP1, GABPA, LDOC1, RBMS3, LIF, GSDMD, IFNL4, MAOA, TCHP, GNRH1, COMP, AGTR1, TNFRSF8, CCNB1, WT1, CYP3A4, NGFR, ABCA1, TGFA, CR1, FOXO1, DNMT1, GUCY1A2, RABEPK, TATDN1, SNORD78, TNFSF10, CD28, SELL, EPCAM, HDAC1, ASCC1, TOP2A, IFI44L, LMNA, CYP2D6, MGMT, AKR1B1, CHN2, RASGRF1, EP300, IL10, LEPR, ABCC1, MAP2K1, FGF23, IGHJ6, REN, TRIM25, TNC, PRH2, GSTM1, SCG5, RUNX1, CCL4, MANEA, SHH |

Table S.2: COVID-19 related genes with more than 5 references (based on DisGeNET database)

| **Genes** |
| --- |
| ACE2, CRP, IL6, ACE, REN, TNF, TMPRSS2, CD4, CD8A, AGT, ALB, IL1B, GPT, F2, IL10, LOC102724971, LOC102723407, INS, IFNG, CXCL8, AMH, IL1A, IFNA1, FURIN, CSF2, DPP4, TNNI3, F3, IL2, GOT1, CRX, IFNB1, NFKB1, AGTR1, RPGR, PDB1, IL6R, PLAT, CALCA, CCL2, IL17A, SERPINA13P, HLA-C, LSAMP, LAMP3, ABO, HBA1, CENPJ, IL2RA, NELFCD, MB, CXCL10, VWF, CTSL, NLRP3, CTRL, CTSB, IL4, SPECC1, MAS1, ZFYVE9, MS4A1, PDCD1, RTN1, RTN4, BSG, MTOR, NCKIPSD, AHI1, SOAT1, VEGFA, PLG, KRT20, F8, JAK1, SERPINA5, CD19, IFNA2, PSMD1, STAT3, TTR, KNG1, NPPB, EMSLR, AGTR2, CSF3, IL7, IL18, PROS1, LINC01672, ESR1, NFE2L2, PSS, C5, EEF1A2, ERBB2, G6PD, GGT1, NCAM1, ADAM17, TLR4, GGTLC5P, GGTLC3, GGT2, GGTLC4P, FCGR3A, FCGR3B, HSPA5, IL1RN, INSRR, JAK2, CCL3, CCL5, TLR3, KLK4, SH2D3C, BTK, VPS51, ABCB1, WDTC1, PGR-AS1, C3, CD14, F10, HIF1A, HLA-A, SERPINF2, SLC5A2, ADAMTS2, TLR7, STS, CYP3A4, FLT4, HMGB1, LTF, EPCAM, COX2, PRF1, MAPK1, PKD2L1, SLC33A1, ADAMTS13, IGKV2D-29, PYCARD, ASZ1, AR, CASP1, CDSN, CEL, HLA-B, IGHE, IL13, LCT, MME, MUC1, OCA2, PRKAA2, STAT1, B3GALNT1, RHOD, SCPEP1, FUZ, SCAI, MS2, AKT1, C5AR1, CASP3, CD48, CD68, EGFR, DMTN, EPHA3, EPO, ICAM1, JUN, MBL2, MEFV, MPO, PRKAA1, PRKAB1, NECTIN1, SELP, TFPI, APOL1, RNMT, HGS, ISG15, CABIN1, CD2AP, CD274, IL23A, CDCA7L, OTOR, IFIH1, SLTM, OMA1, SLC6A19, SERPINA3, ACR, PARP1, AGER, ANGPT1, ANPEP, AQP4, CD38, CDKN3, CCR5, CRYGC, CYP2D6, ATN1, ELANE, FBL, GLP1R, HLA-DRB1, HMOX1, MET, NR3C2, MMP9, MUC5AC, MX1, NEU1, OXT, SERPINE1, PIK3CA, PIK3CB, PIK3CD, PIK3CG, PPARG, PROC, MOK, SAA1, NRP1, NAPSA, SH2D3A, IFITM3, PITRM1, COPE, DDX58, PLA2G15, NAAA, BBS9, FAM3B, ACSS2, ACCS, CRYGEP, A1BG, KLK3, FAS, CD40LG, CECR, CPOX, CSF1, SLC25A10, DAPK3, DHODH, DIO3, EPHA2, FUT3, GCG, NR3C1, HBB, HBG2, CFI, IGF1, ISG20, ITGAM, IVD, KLRC1, LEP, LRP2, MMP8, NOS2, NPC1, MAPK8, PTGS2, PTPRC, TF, UMOD, AD5, SKAP2, PPIG, RAPGEF5, CTPP, CLEC4M, CPQ, SLC27A5, SPACA9, MCF2L, SIRT1, SETD2, FEV, PARP9, ZGPAT, GGTLC1, COPD, LOC102724197 |

Table S.3: Proteins with a degree greater than 50 in the network were obtained from the STRING database

|  | Proteins with a degree greater than 50 |
| --- | --- |
| COVID-19 infection-related proteins | ABCB1, ACE, ACE2, ADAM17, ADIPOQ, AGER, AGT, AGTR1, AKT1, ALB, ANPEP, APOE, APP, AR, BCL2L1, BRCA1, BRCA2, BTK, C3, CALCA, CASP1, CASP3, CASP8, CASP9, CAT, CAV1, CCL2, CCNA2, CCNB1, CCND1, CCR5, CD14, CD19, CD2, CD209, CD274, CD28, CD34, CD38, CD4, CD40, CD40LG, CD44, CD68, CD80, CD86, CD8A, CDC42, CDH1, CDH17, CDK1, CDK2, CDK4, CDKN1A, CFTR, CNR1, CREB1, CSF1, CSF2, CSF3, CTNNB1, CTSB, CTSD, CXCL10, CXCR1, CXCR4, CYBB, CYCS, CYP3A4, DNMT1, DPP4, EDN1, EGF, EGFR, EGR1, ELANE, ENG, EP300, EPCAM, EPHA2, EPO, ERBB2, ESR1, EZH2, F2, F3, FAS, FASLG, FGF2, FLT3, FLT4, FN1, FOS, FOXO1, FOXP3, FURIN, GAPDH, GCG, GDNF, GJA1, HDAC1, HGF, HIF1A, HMGB1, HMOX1, HP, HRAS, HSP90AA1, HSPA5, HSPB1, HSPD1, ICAM1, IFNB1, IFNG, IGF1, IGF2, IL10, IL18, IL1A, IL1B, IL2, IL23A, IL2RA, IL2RG, IL4, IL6, IL6R, INS, INSR, IRF3, IRS1, ITGAL, ITGAM, ITGB2, ITIH4, JAK1, JAK2, JUN, KDR, KIT, KLK3, KRAS, LDLR, LEP, LEPR, LIF, MAP2K1, MAPK1, MAPK8, MAPT, MBP, MCL1, MET, MGMT, MME, MMP1, MMP13, MMP2, MMP3, MMP8, MMP9, MTOR, MX1, MYCN, NCAM1, NFE2L2, NFKB1, NGFR, NLRP3, NOS1, NR3C1, NRP1, OCLN, PARP1, PDCD1, PF4, PIK3CA, PIK3CB, PIK3CD, PIK3CG, PLAT, PLAU, PLG, POMC, POU5F1, PPARG, PPIG, PRL, PROM1, PSEN1, PTEN, PTGS2, PTH, PTK2B, RB1, RELA, REN, RET, RHOA, SELL, SELP, SERPINE1, SHH, SIRT1, SLC2A1, SMAD2, SMAD3, SMAD4, SOD1, SOD2, SOX2, SOX9, SP1, SPP1, SQSTM1, STAT1, STAT3, STAT5A, STAT5B, SYP, TERT, TFRC, TGFB1, TIMP1, TIMP2, TLR3, TLR4, TLR7, TLR8, TNF, TNFSF11, TP53, TWIST1, TXN, VEGFA, VIM, VTN, WT1, YWHAZ |
| Other proteins | ACHE, ACTB, AFP, ANGPT1, ANXA5, APOA1, APOB, BDNF, BGLAP, CCL3, CCL4, CCL5, CCL7, CDKN2A, CRP, CTGF, CTLA4, CXCL12, CXCL8, CYP19A1, CYP2E1, DDX58, ELN, ENO2, FCGR3A, FCGR3B, GFAP, GPT, GRIN2B, HPRT1, HTT, IFIH1, IFNA1, IGF1R, IGFBP3, IL13, IL17A, IL1RN, IL3, IL7, ISG15, KITLG, KNG1, KRT19, LMNA, LPL, MDM2, MPO, NES, NF1, NOS2, NOS3, NOTCH1, NPY, NRAS, NT5E, NTF3, PECAM1, PPARA, PRF1, PTPN11, PTPRC, RAC1, SAA1, SST, TAC1, TCHP, TH, TNFSF10, TTR, VWF |

Table S.4: Details of each of drug-gene interactions

| Gene | Drug Name | KEGG Pathway | Structure ID | Target Class | Accession | Action Value | Action Type |
| --- | --- | --- | --- | --- | --- | --- | --- |
| **AKT1** | miltefosine | Acute myeloid leukemia | 1810 | Kinase | P31749 | 5.02 | IC50 |
| **AKT1** | niclosamide | Acute myeloid leukemia | 1912 | Kinase | P31749 | 4.23 | IC50 |
| **AKT1** | quercetin | Acute myeloid leukemia | 3514 | Kinase | P31749 | 5.27 | IC50 |
| **AKT1** | midostaurin | Acute myeloid leukemia | 5231 | Kinase | P31749 | 6.02 | Kd |
| **NFKB1** | bortezomib | Acute myeloid leukemia | 391 | Unclassified | P19838 | 8.01 | IC50 |
| **NFKB1** | sitosterol | Acute myeloid leukemia | 2451 | Cytosolic other | P19838 | 5.6 | IC50 |
| **NFKB1** | cepharanthine | Acute myeloid leukemia | 5375 | Cytosolic other | P19838 |  | INHIBITOR |
| **STAT3** | digitoxin | Acute myeloid leukemia | 881 | Transcription factor | P40763 | 6.15 | IC50 |
| **STAT3** | digoxin | Acute myeloid leukemia | 882 | Transcription factor | P40763 | 5.73 | IC50 |
| **STAT3** | niclosamide | Acute myeloid leukemia | 1912 | Transcription factor | P40763 | 6.6 | IC50 |
| **STAT3** | nifuroxazide | Acute myeloid leukemia | 1928 | Transcription factor | P40763 | 5.52 | EC50 |
| **STAT3** | ouabain | Acute myeloid leukemia | 2004 | Transcription factor | P40763 | 5.94 | IC50 |
| **RELA** | bortezomib | Acute myeloid leukemia | 391 | Unclassified | Q04206 | 8.01 | IC50 |
| **TNF** | thalidomide | Adipocytokine signaling pathway | 2616 | Cytokine | P01375 | 8 | IC50 |
| **TNF** | lenalidomide | Adipocytokine signaling pathway | 3317 | Cytokine | P01375 | 7.886 | IC50 |
| **TNF** | adalimumab | Adipocytokine signaling pathway | 4904 | Cytokine | P01375 |  | ANTIBODY BINDING |
| **TNF** | golimumab | Adipocytokine signaling pathway | 4967 | Cytokine | P01375 |  | ANTIBODY BINDING |
| **TNF** | certolizumab pegol | Adipocytokine signaling pathway | 4968 | Cytokine | P01375 | 10.046 | ANTIBODY BINDING |
| **TNF** | infliximab | Adipocytokine signaling pathway | 4974 | Cytokine | P01375 |  | ANTIBODY BINDING |
| **TNF** | etanercept | Adipocytokine signaling pathway | 4978 | Cytokine | P01375 |  | INHIBITOR |
| **TNF** | plecanatide | Adipocytokine signaling pathway | 5208 | Cytokine | P01375 | 8.42 | Kd |
| **IL6** | prednisolone | African trypanosomiasis | 2245 | Cytokine | P05231 | 8.38 | IC50 |
| **IL6** | raloxifene | African trypanosomiasis | 2351 | Cytokine | P05231 | 8.05 | IC50 |
| **IL6** | siltuximab | African trypanosomiasis | 4977 | Cytokine | P05231 |  | ANTIBODY BINDING |
| **IL1B** | canakinumab | African trypanosomiasis | 4952 | Cytokine | P01584 |  | ANTIBODY BINDING |
| **IL1B** | rilonacept | African trypanosomiasis | 5105 | Cytokine | P01584 | 12.301 | INHIBITOR |
| **GAPDH** | adenosine | Alzheimer disease | 90 | Enzyme | P04406 | 4.46 | IC50 |
| **ADAM17** | prednisolone | Alzheimer disease | 2245 | Unclassified | P78536 | 7.83 | IC50 |
| **VEGFA** | acetylcysteine | Bladder cancer | 66 | Secreted | P15692 |  |  |
| **VEGFA** | pegaptanib | Bladder cancer | 4905 | Secreted | P15692 |  | ANTAGONIST |
| **VEGFA** | aflibercept | Bladder cancer | 4916 | Secreted | P15692 | 12.301 | INHIBITOR |
| **VEGFA** | bevacizumab | Bladder cancer | 4956 | Secreted | P15692 |  | ANTIBODY BINDING |
| **VEGFA** | ranibizumab | Bladder cancer | 4971 | Secreted | P15692 |  | ANTIBODY BINDING |
| **VEGFA** | brolucizumab | Bladder cancer | 5363 | Secreted | P15692 | 8.8 | INHIBITOR |
| **IFNAR2** | peginterferon beta-1a | Cytokine-cytokine receptor interaction | 5137 | Membrane receptor | P48551 |  | AGONIST |
| **IFNAR2** | interferon beta-1a | Cytokine-cytokine receptor interaction | 5139 | Membrane receptor | P48551 |  | AGONIST |
| **IFNAR2** | interferon beta-1b | Cytokine-cytokine receptor interaction | 5140 | Membrane receptor | P48551 |  | AGONIST |
| **IFNAR2** | peginterferon alfa-2a | Cytokine-cytokine receptor interaction | 5158 | Membrane receptor | P48551 |  | AGONIST |
| **IFNAR2** | peginterferon alfa-2b | Cytokine-cytokine receptor interaction | 5164 | Membrane receptor | P48551 |  | AGONIST |
| **IFNAR2** | ropeginterferon alfa-2b | Cytokine-cytokine receptor interaction | 5338 | Membrane receptor | P48551 |  | BINDING AGENT |
| **IFNAR1** | peginterferon beta-1a | Cytokine-cytokine receptor interaction | 5137 | Membrane receptor | P17181 |  | AGONIST |
| **IFNAR1** | interferon beta-1a | Cytokine-cytokine receptor interaction | 5139 | Membrane receptor | P17181 |  | AGONIST |
| **IFNAR1** | interferon beta-1b | Cytokine-cytokine receptor interaction | 5140 | Membrane receptor | P17181 |  | AGONIST |
| **IFNAR1** | peginterferon alfa-2a | Cytokine-cytokine receptor interaction | 5158 | Membrane receptor | P17181 |  | AGONIST |
| **IFNAR1** | peginterferon alfa-2b | Cytokine-cytokine receptor interaction | 5164 | Membrane receptor | P17181 |  | AGONIST |
| **IFNAR1** | ropeginterferon alfa-2b | Cytokine-cytokine receptor interaction | 5338 | Membrane receptor | P17181 |  | BINDING AGENT |
| **IL6R** | tocilizumab | Cytokine-cytokine receptor interaction | 4933 | Membrane receptor | P08887 |  | ANTIBODY BINDING |
| **IL6R** | sarilumab | Cytokine-cytokine receptor interaction | 5237 | Membrane receptor | P08887 | 11.1 | ANTIBODY BINDING |
| **IL6ST** | tocilizumab | Cytokine-cytokine receptor interaction | 4933 | Membrane receptor | P40189 |  | ANTIBODY BINDING |
| **IL6ST** | oprelvekin | Cytokine-cytokine receptor interaction | 5026 | Membrane receptor | Q14626 |  | AGONIST |
| **IL6ST** | sarilumab | Cytokine-cytokine receptor interaction | 5237 | Membrane receptor | P40189 | 11.1 | ANTIBODY BINDING |
| **JAK1** | sunitinib | Hepatitis C | 2544 | Kinase | P23458 | 8.05 | ANTAGONIST |
| **JAK1** | crizotinib | Hepatitis C | 4187 | Kinase | P23458 | 6.48 | INHIBITOR |
| **JAK1** | ruxolitinib | Hepatitis C | 4190 | Kinase | O60674 | 6.53 | IC50 |
| **JAK1** | tofacitinib | Hepatitis C | 4713 | Kinase | O60674 | 7.12 | INHIBITOR |
| **JAK1** | ceritinib | Hepatitis C | 4866 | Kinase | P23458 | 5.43 | IC50 |
| **JAK1** | nintedanib | Hepatitis C | 4903 | Kinase | P23458 | 8.32 | Kd |
| **JAK1** | baricitinib | Hepatitis C | 5202 | Kinase | P23458 | 8.2291 | INHIBITOR |
| **JAK1** | midostaurin | Hepatitis C | 5231 | Kinase | P23458 | 6.17 | Kd |
| **JAK1** | upadacitinib | Hepatitis C | 5346 | Kinase | P23458 | 7.33 | INHIBITOR |
| **JAK1** | fedratinib | Hepatitis C | 5347 | Kinase | P23458 | 6.97 | INHIBITOR |


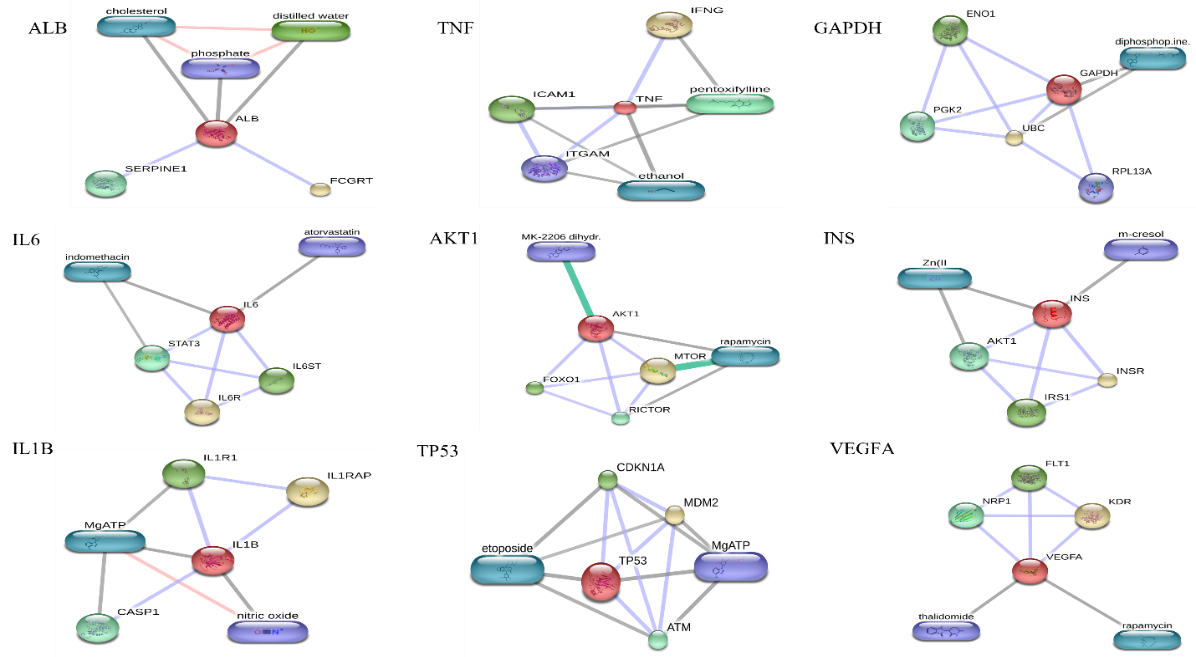


**Fig S.1:** five chemical compounds corresponding to each of the 9 hub proteins


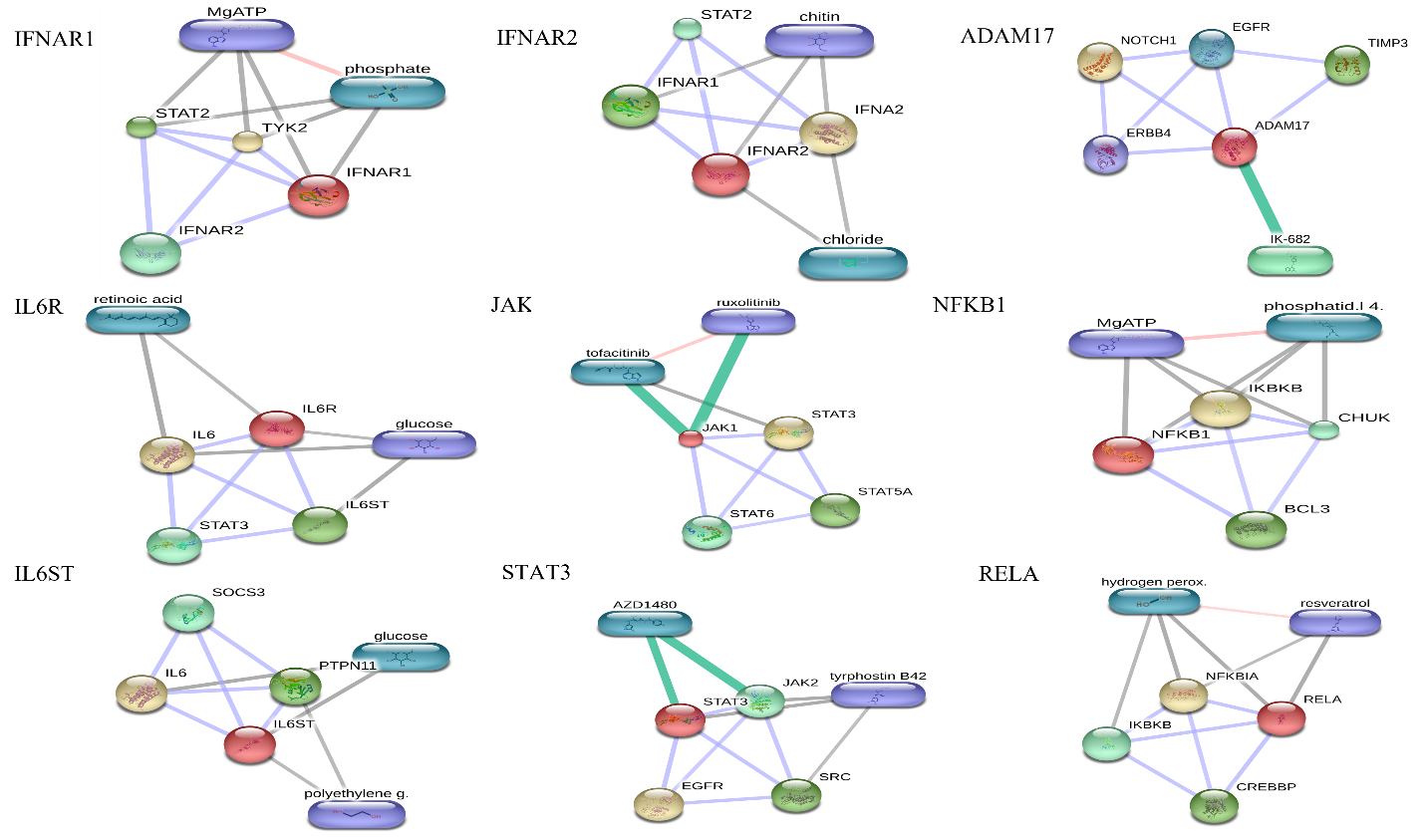


**Fig S.2:** five chemical compounds corresponding to each of the 9 driver genes
